# Moderators of treatment outcomes for LGBTQ+ military veterans in the PRIDE in All Who Served health promotion group

**DOI:** 10.1371/journal.pone.0282376

**Published:** 2024-11-27

**Authors:** Michelle M. Hilgeman, Robert J. Cramer, Andréa R. Kaniuka, Ryan A. Robertson, Teddy Bishop, Sarah M. Wilson, Heather A. Sperry, Tiffany M. Lange

**Affiliations:** 1 Research & Development Service, Tuscaloosa VA Medical Center, Tuscaloosa, Alabama, United States of America; 2 Department of Psychology, The University of Alabama, Tuscaloosa, Alabama, United States of America; 3 Public Health Department, National Opinion Research Center (NORC), University of Chicago, Chicago, Illinois, United States of America; 4 VA Health Services Research & Development Center of Innovation to Accelerate Discovery and Practice Transformation (HSR&D ADAPT COIN), Durham VA Healthcare System, Durham, North Carolina, United States of America; 5 Department of Psychiatry and Behavioral Sciences, Duke University School of Medicine, Durham, North Carolina, United States of America; 6 LGBTQ+ Health Program, Veteran Health Indiana, Indianapolis, Indiana, United States of America; 7 Medical Affairs, Defense Health Agency, Falls Church, Virginia, United States of America; University of Central Florida, UNITED STATES OF AMERICA

## Abstract

**Background:**

Veterans who identify as lesbian, gay, bisexual, transgender, queer, questioning, and related identities (LGBTQ+) have faced discrimination that puts them at increased risk for depression, anxiety, and suicide. Upstream interventions like the PRIDE in All Who Served program can improve internalized prejudice, suicidality, symptoms of depression, and symptoms of anxiety by addressing minority stress, facilitating social connection, and promoting engagement with the healthcare system. Yet, little is known about who benefits most from these types of services.

**Methods and materials:**

Sixty-six US military veterans (Mean age = 47.06, SD = 13.74) provided outcome surveys before and after a 10-week health promotion group for LGBTQ+ individuals at one of 10 Veterans Health Administration (VA) Medical Centers. Subscales of a coping self-efficacy measure (e.g., problem-solving, social support, thought-stopping), and demographic factors were examined as moderators of treatment outcomes.

**Results:**

Coping self-efficacy moderated effects across treatment outcomes with those lower in coping self-efficacy beliefs reporting the greatest benefit of the intervention. Reduction in anxiety symptoms was moderated only by problem-solving coping self-efficacy, while suicidality was moderated only by social support. Reduction of internalized prejudice and depression symptoms were moderated by both problem-solving and social support coping self-efficacy, while thought-stopping (a frequent target of traditional cognitive therapies) only moderated internalized prejudice, but not clinical symptom indicators. Most demographic factors (e.g., age, race, gender) did not impact treatment outcomes; however, sexual orientation was significant such that those who identified as bisexual, queer, or something else (e.g., pansexual) had greater reductions in internalized prejudice than their single gender-attracted peers.

**Discussion and conclusion:**

Individual differences like coping self-efficacy and sexual orientation are rarely considered in clinical care settings when shaping policy or implementing tailored programs. Understanding implications for who is most likely to improve could inform program refinement and implementation of affirming interventions for minoritized people.

## Introduction

Who improves from an intervention is a critical question for clinical program evaluation and intervention refinement in healthcare settings [1]. This paper examines potential moderators of treatment outcomes for the PRIDE in All Who Served health promotion group for military veterans who identify as lesbian, gay, bisexual, transgender, queer, questioning and related identities (LGBTQ+) in the U.S. Department of Veterans Affairs healthcare system (VA) [2,3]. Examining differences in response to this new and spreading program has potential implications for implementation at both the local clinician level and for leadership tasked with meeting needs of an increasingly visible LGBTQ+ veteran population choosing the VA as their primary place to access health care [4].

### Health disparities observed in LGBTQ+ military veterans

LGBTQ+ veterans are a marginalized group within the VA [5]. Available estimates suggest there are more than one million veterans who identify as lesbian, gay, or bisexual and approximately 130,000 veterans who identify as transgender or gender diverse [5]. Yet, reports from active military service members point to even higher rates, with 6.1% identifying as lesbian, gay, or bisexual in 2015 [6].

LGBTQ+ veterans face systemic discrimination and other unique barriers to healthcare that impact their access to care [7]. Inequities in mental health outcomes include higher rates of suicidal ideation and attempts, post-traumatic stress disorder (PTSD), and depression compared to the general veteran population [8,9]. Worse mental health outcomes are due, in large part, to minority stress [10,11] which includes: (a) experiences of rejection, discrimination, harassment, and victimization, and (b) internalized stigma, homophobia, biphobia, and/or transphobia. For LGBTQ+ veterans, minority stress stems from both within and outside of the military setting [7,12,13]. These specific minority stressors include the legacy of the Don’t Ask, Don’t Tell policy [14] which was repealed just over a decade ago and banned sexual minorities from serving openly in the military [15], as well as challenges faced by transgender service members and veterans with military bans being lifted and reinstated several times between 2016 and 2021 [16,17]. These policies, while currently rescinded, contribute to a military culture that leads to concealment of LGBTQ+ identity [18], an established contributor to negative mental health outcomes among LGBTQ+ people (e.g., depression, anxiety) [19,20].

### Health-promotion interventions for LGBTQ+ veterans

Tailored programs that bolster resilience and address the impact of minority stress on LGBTQ+ veterans are emerging in mental health clinics and wellness-focused programs in healthcare settings [9]. The PRIDE in All Who Served program is an affirmative care intervention developed for delivery outside of mental health settings to promote health for LGBTQ+ veterans at the VA [2]. See Table 1. Ten weeks of content addresses LGBTQ+-related identity resilience and stress (e.g., continuums of identity, identity development, coming out/emergence), enhance health literacy and engagement with services (e.g., sexual health, affirmative care, whole health), increases social connection (e.g., health and safety in relationships, LGBTQ+ community resources), and processes minority stress associated with military service experience (e.g., military culture, VA culture). Each session is guided by participant handouts and a manual for group facilitators that includes information on establishing an affirmative environment in the session and more broadly at the facility. Mental health-related diagnoses and related distress are not required for Veterans to access the PRIDE in All Who Served group. Yet, initial pre-post evaluations show reduction in veteran symptoms of distress (e.g., suicidal ideation) and improvement in identity-related resilience like self-acceptance [2,3]. Implementation support is also provided, resulting in positive impacts on facility-level Healthcare Equality Index scores and rapid spread beyond the development site in just five years [3,21].

**Table 1:** Pride in All Who Served topic covered each week.

Week	Session Topic
1	Continuums of identity, LGBTQ+ terminology and definitions
2	Coming out; emergence and disclosure
3	Identity models
4	US military culture, then & now
5	VA culture–the changes ahead
6	Affirmative care; whole health
7	Sexual health
8	Healthy intimate relationships; safety in relationships
9	LGBTQ+ families
10	Community resources & conclusion

Note. See Lange and colleagues development and feasibility work [2] for detailed information about the content and veteran handouts used in each session.

### Possible moderators of LGBTQ+ intervention outcomes

The clinical utility of identifying non-diagnostic individual differences that may impact treatment response has received considerable attention [22–24]. However, very few studies have examined treatment moderators for interventions created for LGBTQ+ persons. Connecting to the LGBTQ+ community, seeking social support, and coping strategies like cognitive reframing and emotion regulation can buffer the linkage between minority stress and negative mental health [25–27]. The mixed results of prior studies and gaps in empirical literature characterize the current vacuum in which clinicians and healthcare leaders are making treatment delivery decisions for LGBTQ+ individuals in their clinics.

Some demographic variables such as age, gender, and race/ethnicity are widely studied moderators of treatment in mental health outcomes research [28,29]. For example, some review papers have indicated that culturally adapted interventions are more effective when provided to homogenous groups of individuals rather than combining those with mixed cultural, racial, or ethnic backgrounds [30–32], while other individual factors like age and gender have been less consistent. For example, in two studies of PTSD treatment, one found no impact of age while another found an effect of gender with women reporting greater improvement compared to men [33,34]. Note, we are using language reported in original papers to describe research samples (i.e., “men” and “women” rather than “cisgender men” and “cisgender women”) where gender identity and experience are not recorded.

### Coping self-efficacy

Coping self-efficacy (CSE)–defined as the beliefs about one’s ability to use coping strategies in the face of aversive experiences–has been linked to mental health and identity-related outcomes in both general population and LGBTQ+ samples [35]. Collective evidence suggests CSE and related strategies may be associated with better mental health for LGBTQ+ populations [36–38]. Specifically, coping and resilience appear to buffer the negative impacts of stigma for LGBTQ+ people and are logical mechanisms of focus for intervention [36–38]. CSE has also been identified as a mechanism of therapeutic change following cognitive behavioral group therapy for social anxiety disorder (CBT) [39]. After 12-months of group CBT treatment, participants reported greater cognitive reappraisal self-efficacy (i.e., akin to stopping negative thoughts), which was associated with lower levels of social anxiety symptoms. Among LGBTQ+ adults seeking care at an urban Federally Qualified Health Center, lower CSE has been associated with identity concealment and uncertainty about identity, including a decreased belief in the ability to engage in problem-focused coping, thought stopping, or asking for social support [40]. Similarly, expectations of interpersonal rejection were related to lower levels of each CSE subscale while higher internalized homonegativity was associated with lower beliefs about using problem-focused coping or ability to receive social support from others in a community-based SM people [41].

A similar pattern of association between domains of coping-self efficacy and improved mental health has been observed in military active-duty samples. Lower levels of thought stopping, problem-focused coping, and asking for social support were linked with greater stress, anxiety, depression, and PTSD symptoms, as well as poorer overall physical health [42]. The same study noted greater thought stopping beliefs were associated with reduced likelihood of lifetime attempts as well as future suicide attempts. Coping self-efficacy beliefs may be of particular importance for veterans due to prior combat-related stressors, such as prolonged threats of danger, isolation from support systems at home, and the residual impacts of LGBTQ+ military bans which complicate efforts to find community with other LGBTQ+ veterans [43,44]. Additionally, access to social support and CSE have been described as protective factors in decreasing PTSD symptoms and overall psychological distress in general veteran population samples [45–47]. Altogether, coping self-efficacy may be a valuable individual difference worth examining within the context of group-based interventions–particularly for LGBTQ+ individuals in clinical settings.

### The present study

Very few LGBTQ+-affirming programs for military veterans exist, particularly with evaluation information that includes potential mechanisms (e.g., identity-related resilience) [48]. Yet replicability of high-quality programs and data-driven decision-making are core features of a learning healthcare system like VA [49]. PRIDE in All Who Served [2] is a group protocol for LGBTQ+ veterans in the VA with an emerging evidence base [3,21]. This paper examines clinical program evaluation data from the PRIDE in All Who Served program to identify potential moderators of program outcomes including internalized stigma and related mental health symptoms (i.e., depression, anxiety, and suicidal ideation). Evidence of program outcome moderators may offer opportunities to further improve health promotion programs for LGBTQ+ veterans and identify those who may benefit most. Therefore, we explored the following questions using clinical program evaluation data:

1. We assessed whether coping self-efficacy would moderate PRIDE in All Who Served outcomes. We hypothesized that those with lower levels of CSE would have greater reductions in internalized prejudice and mental health symptoms over program participation.
2. We assessed whether demographic factors (i.e., race/ethnicity, age, sexual orientation, and gender) would moderate PRIDE in All Who Served outcomes. Given the lack of prior literature on this topic, these analyses were exploratory with no set hypotheses.

## Materials and method

### Participants

Table 2 contains sample demographic and individual difference descriptive statistics. Participants attended PRIDE in All Who Served groups in 2018–2019. Race was primarily White (47%), Black (37.9%), or Multiracial/Biracial (9.1%). Sexual orientation, gender identity, and military service branch varied considerably within the sample. The group was primarily of non-Hispanic ethnicity, with an average middle-adult age. Pre-program depressive symptom mean scores were in the moderate range, and post-program scores were in the mild range [50]. Both pre- and post-program anxiety symptom average scores were in the mild range [51]. Pre-program suicidality average scores fell between “no chance at all” and “rather unlikely,” whereas post-program scores reflected a response of “no chance at all” [52]. Both pre- and post-program internalized prejudice mean scores reflected an average response of “strongly disagree” [53]. Though typical ranges for coping self-efficacy are not established, problem-solving and thought stopping coping self-efficacy average scores were approximately equal to those observed in active-duty treatment-seeking samples. Additionally, the current sample of LGBTQ+ veterans’ perceptions of their ability to obtain social support was lower compared to published active-duty treatment-seeking samples [42].

**Table 2:** Veteran demographic and program outcome descriptive information.

Variable	N (%)
Race (N = 66)	
White	31 (47.0)
Black	25 (37.9)
Filipino	1 (1.5)
Bi- or multiracial	6 (9.1)
Other minority (unspecified)	3 (4.5)
Ethnicity (N = 64)	
Not of Hispanic/Latino or Spanish origin	62 (93.9)
Puerto Rican	1 (1.5)
Other Hispanic/Latino(a) Origin	1 (1.5)
Missing	2 (3.0)
Sexual orientation (N = 65)	
Gay	19 (28.8)
Lesbian	11 (16.7)
Queer	4 (6.1)
Straight	4 (6.1)
Questioning	2 (3.0)
Pansexual	5 (7.6)
Bisexual	4 (6.1)
Don’t know	4 (6.1)
Other sexual minority	9 (13.6)
Multiple identities	3 (4.5)
Missing	1 (1.5)
Gender identity (N = 64)	
Cisgender male	21 (31.8)
Cisgender female	19 (28.8)
Transgender male	6 (9.1)
Transgender female	13 (19.7)
Gender Queer	2 (3.0)
Multiple identities	3 (4.5)
Missing	2 (3.0)
Military branch (N = 66)	
Army	37 (56.1)
Air Force	8 (12.1)
Navy	13 (19.7)
Marines	6 (9.1)
Coast Guard	2 (3.0)
	*M* (*SD*)
Age (N = 65)	47.06 (13.74)
Pre-program internalized prejudice (N = 61)	7.21 (4.73)
Post-program internalized prejudice (N = 60)	6.69 (4.04)
Pre-program depressive symptoms (N = 60)	10.77 (7.03)
Post-program depressive symptoms (N = 57)	9.18 (6.23)
Pre-program anxiety symptoms (N = 60)	8.76 (6.20)
Post-program anxiety symptoms (N = 57)	7.33 (5.20)
Pre-program suicidality (N = 66)	1.54 (1.49)
Post-program suicidality (N = 62)	1.00 (1.25)
Problem-solving coping self-efficacy (N = 66)	37.24 (13.88)
Thought stopping coping self-efficacy (N = 66)	20.46 (10.58)
Getting social support coping self-efficacy (N = 66)	15.24 (8.50)

Notes. N = Sample size; M = Mean; SD = Standard deviation.

### Procedure

The PRIDE in All Who Served program was funded by the VA Innovation Ecosystem as an innovation investment program. From October 2017 to September 2019, ten VA sites participated in data collection efforts for program evaluation and quality improvement purposes. Procedures were reviewed by the Tuscaloosa VA Medical Center Institutional Review Board (Project title: [1316792–1] *Serving All Who Served*: *Improving Access to Health Care for LGBT Veterans*; IRB Reference #: 00254/19-01) and the VA Central Office program sponsor consistent with federal regulations (e.g., VA Program Guide 1200.21: Veterans Health Administration Operations Activities That May Constitute Research). LGBTQ+ veterans were identified by group facilitators at each site via self-referrals and referrals from other VA providers. A pre/post single group design was used to assess veteran-level changes across the 10-week program. Veteran participants at each site completed a voluntary paper and pencil questionnaire during the first group session (session 1) and last group session (session 10). Informed consent was completed as a verbal discussion in a group setting (during the 1^st^ and 10^th^ sessions of the 10-week group), with written instructions presented on the first page of the paper survey packets. Outcome questionnaires were collected anonymously using a participant-created ID consisting of letters and numbers to connect assessments over time. Questionnaires were distributed and collected by group facilitators from each site who then scanned and returned them to the evaluation team via encrypted email. Participants who only completed the pre- or post-questionnaire were excluded from analyses.

### Measures

#### Demographics

Participants completed a short demographic form which included information such as sexual orientation, gender identity, sex assigned at birth, age, race, ethnicity, and military service branch. Extensive gender and sexual orientation identity label list choices were provided along with the option to write-in responses. In this way, LGBTQ+ veterans were able to express their unique demographic-related identities within the scope of participating in PRIDE in All Who Served, see [2,3] for more information about the evaluation approach.

#### Coping self-efficacy

The Coping Self-Efficacy Scale (CSES) [35] is a 13-item self-report questionnaire which assesses confidence in engaging in three forms of coping: (a) problem-focused coping (e.g., finding solutions), (b) stopping unpleasant thoughts/emotions (e.g., keeping from feeling sad), and (c) using social support (e.g., making new friends). Items are rated on an 11-point Likert scale, ranging from 0 (*cannot do at all*) to 10 (*certain can do*). Subscale scores are generated for each subcomponent via summation: problem-focused coping (total of 6 items), thought stopping (total of 4 items) and social support (3 items); higher scores indicate greater confidence in coping. Subscale internal consistencies among a prior active-duty military sample ranged from good to excellent (problem-focused coping: *α* = .94; thought stopping: *α* = .89; social support: *α* = .82; [42]. Internal consistency in the current study was also high (problem-focused coping: α = .92; thought stopping: α = .94; social support: α = .82).

#### Depressive symptoms

The Patient Health Questionnaire-9 (PHQ-9) [50] is a 9-item self-report questionnaire used to assess DSM-IV depression criteria, such as lack of energy and difficulty sleeping. Items are scored on a 4-point Likert scale ranging from 0 (*not at all*) to 3 (*nearly every day*). Total scores are generated via summation, with higher scores indicating greater presence of depressive symptoms. Scores range from 0 to 27, with the following classifications: 0–4: minimal; 5–9: mild; 10–14: moderate; 15–19: moderately severe; and 20–27: severe. A clinical cut-off score can also be used, with scores greater than 10 indicating clinically elevated risk of major depression. In the current study, the total score was used. The PHQ demonstrates good internal consistency veteran samples (*α* = .86) [54]; the internal consistency in the current study was also acceptable for pre- (*α* = 88) and post-program (*α* = 88) scores.

#### Anxiety symptoms

The Generalized Anxiety Disorder-7 (GAD-7) [51] is a 7-item measure of symptoms of generalized anxiety disorder, such as feeling nervous, anxious, or on edge. Items are scored on a 4-point Likert scale ranging from 0 (*not at all*) to 3 (*nearly every day*). Total scores are generated via summation, with higher scores indicating greater presence of anxiety symptoms. Scores range from 0 to 21, with the following classifications: 0–4: minimal; 5–9: mild; 10–14: moderate; 15–21: severe. A clinical cut-off score can also be used, with scores greater than 10 indicating clinically elevated risk of generalized anxiety. In the current study, the total score was used. The GAD-7 demonstrates good internal consistency among veteran samples (α = .89) [55]. The internal consistency in the current study was acceptable for pre- (α = .90) and post-program (α = .90) scores.

#### Suicidality

The Suicide Behaviors Questionnaire-Revised (SBQ-R) [52] is a 4-item measure of suicidality, including: (a) lifetime suicidal thoughts and behaviors, (b) 12-month suicidal ideation, (c) communication of suicidal intent; and (d) likelihood of future suicidal behavior. Total scores are generated via summation, ranging from 3 to 18, with higher scores indicating higher suicidality. A clinical cut-off score can be generated, with scores greater than or equal to 7 representing clinically significant suicide risk. SBQ-R single items are also frequently used individually in order to assess unique aspects of suicidal behavior. In the current study, the last item concerning likelihood of a future suicide attempt was used to operationalize suicidality or suicide risk. The SBQ-R demonstrates excellent internal consistency among veteran samples (*α* = .94) [56]. We did not tabulate internal consistency for the single item used in the current study.

#### Internalized prejudice

The internalized prejudice subscale of the Lesbian, Gay, and Bisexual Identity Scale (LGBIS) [53] is a 27-item measure quantifying 7 aspects of sexual minority identity. For PRIDE in All Who Served program development, the LGBIS was modified to be inclusive of gender diversity through altered instructions. Specifically, the following wording was added: “For those identifying as heterosexual but as gender diverse, please respond to the items on this page only as you feel comfortable in how they may apply to you” (2, p. 493). Items are rated on a six-point Likert scale ranging from 1 (*disagree strongly*) to 6 (*agree strongly*) [53]. The internalized prejudice subscale comprises three items. Internal consistency values were acceptable for pre- (α = .89) and post-program (α = .84) scores.

### Data analysis

Prior to demographic moderation analyses, race, sexual orientation, and gender identity were recoded due to small cell sizes. We acknowledge that collapsing individuals into larger categories is problematic and potentially invalidating; retaining more categories is preferred [57]. The demographics (Table 2) describes the study sample in more detail than was feasible in moderation analyses. Race was reclassified as White (*n* = 31, 47.0%) and racial minority (*n* = 35, 53.0%). In line with sexual identity label literature [58,59], sexual orientation was regrouped as heterosexual/gay/lesbian (i.e., attraction to one sex/gender; *n* = 34, 51.5%) and bisexual/questioning/and related identities (i.e., combining those who identify in ways other than single-sex/gender attraction; *n* = 31, 47.0%). Two step-gender (i.e., items assessing sex assigned at birth and gender identity) was recoded as cisgender male (*n* = 21, 31.8%), cisgender female (*n* = 19, 28.8%), and transgender/gender diverse (*n* = 24, 36.4%) for the moderation analyses. Repeated measures general linear model (GLM) analyses [60,61] were used to examine program moderation analyses. Repeated-measures GLM was selected because the approach: (a) allows for simultaneous inclusion of categorical and continuous variables; (b) enables tests of pre-post program main effects, as well as baseline by pre-post program outcome interaction terms; and (c) provides a flexible set of multivariate and univariate test statistics for interpretation. For all models, the pre-post program outcome was either a mental health symptom (e.g., depressive symptoms) or internalized prejudice. For all models, the moderator was either a coping self-efficacy subscale or demographic variable (e.g., age). For example, for an age by depressive symptoms analysis, the independent variable is the pre-post change in symptoms of depressive symptoms and the moderator is age. We used multivariate (i.e., Wilk’s λ) omnibus tests statistics because doing so reduces likelihood of Type I error. We utilized guidelines for magnitude of effect size interpretation for partial-eta squared values provided by Cohen [62]: small = .01, medium = .06, large = .14. Significant interactions were graphed following procedures recommended by Bauer and Curran [63], including representing low and high levels of continuous moderators at -/+ one standard deviation around the mean.

## Results and discussion

### Coping self-efficacy as a moderator of PRIDE in All Who Served program outcomes

**Fig 1:**
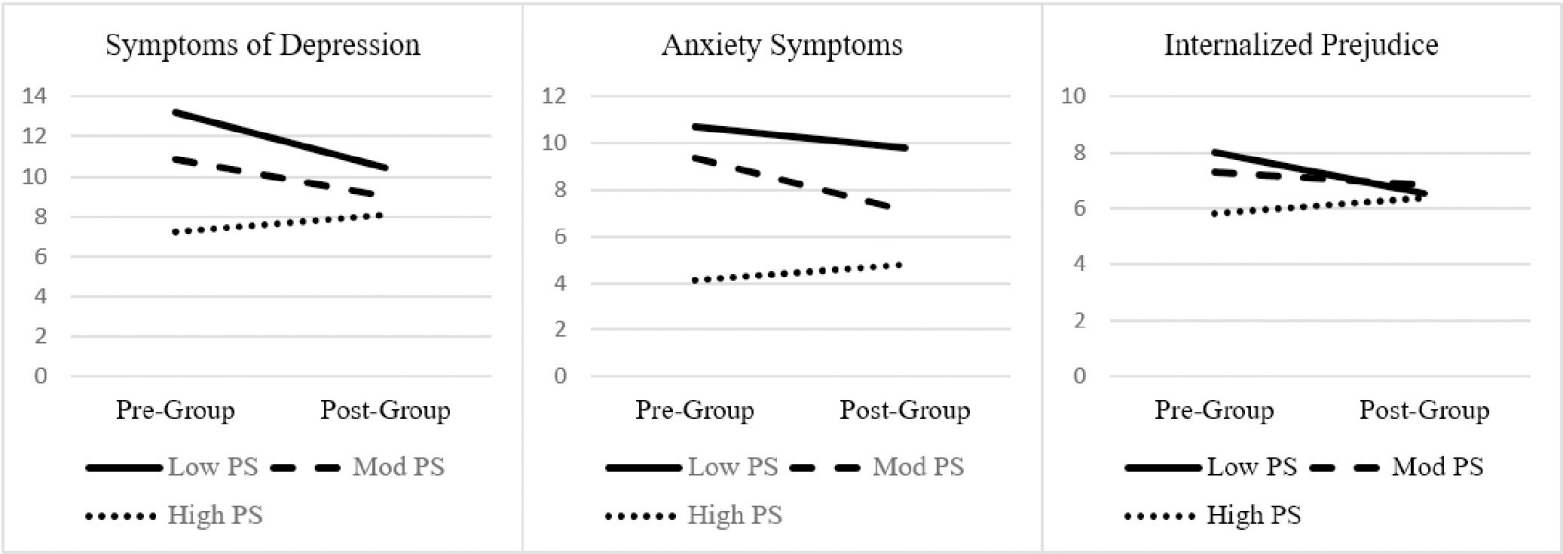
Problem-solving coping-self efficacy as a significant moderator of treatment effect on symptoms of depression, anxiety, and internalized prejudice. Notes. Depression = Patient Health Questionnaire (PHQ-9); Anxiety = Generalized Anxiety Disorder (GAD-7); IP = internalized prejudice subscale of the Lesbian, Gay, and Bisexual Identity Scale (LGBIS); PS = Problem-solving subscale of the Coping Self-Efficacy scale. Test statistics in Table 2.

Table 3 contains test statistics for all CSE subscale moderation analyses. Each CSE subscale was examined across four separate repeated measures GLM analyses, one for each outcome of interest (i.e., depressive symptoms, anxiety symptoms, suicidality, and internalized prejudice). Reviewing Table 3, problem-solving CSE moderated pre-post program reductions in depressive symptoms, anxiety symptoms, and internalized prejudice (all medium effects). Fig 1 depicts the interactions. As predicted, LGBTQ+ veterans lowest in problem-solving CSE reported greater reductions in depressive symptoms and internalized prejudice after attending the PRIDE in All Who Served group. Additionally, LGBTQ+ veterans with moderate problem-solving CSE also reported reductions in depressive and anxiety symptoms.

**Table 3:** Coping self-efficacy subscales as a moderator of treatment effects using repeated measures general linear modeling.

	Depressive Symptoms				Anxiety Symptoms				Suicidality				Internalized Prejudice			
Predictor	Wilks’ λ	F (df)	*p*-value	η_p_^2^	Wilks’ λ	F (df)	*p*-value	η_p_^2^	Wilks’ λ	F (df)	*p*-value	η_p_^2^	Wilks’ λ	F (df)	*p*-value	η_p_^2^
Time	0.84	10.04 (1, 55)	.003	.15***	0.86	9.04 (1, 55)	.004	.14***	0.88	7.98 (1, 60)	.006	.12**	0.89	7.36 (1, 59)	.009	.11**
CSE Problem-solving by time	**0.91**	**5.51** **(1, 55)**	**.023**	**.09** ^**^	**0.92**	4.69 (1, 55)	**.035**	**.08** ^**^	**0.96**	2.69 (1, 60)	.106	.04*	**0.93**	4.39 (1, 59)	**.040**	**.07** ^**^
Time	0.90	6.03 (1, 55)	.017	.10**	0.87	7.87 (1, 55)	.007	.12**	0.83	12.25 (1, 60)	.001	.17***	0.86	9.76 (1, 59)	.003	.14***
CSE Thought Stopping by time	0.97	1.82 (1, 55)	.183	.03*	0.95	2.91 (1, 55)	.094	.05*	0.94	3.90 (1, 60)	.053	.06**	**0.91**	**5.64 (1, 59)**	**.021**	**.09** **
Time	0.82	12.24 (1, 55)	.001	.18***	0.85	9.74 (1, 55)	.003	.15***	0.82	13.08 (1, 60)	.001	.18***	0.86	9.60 (1, 59)	.003	.14***
CSE Social Support by time	**0.90**	**5.84** **(1, 55)**	**.019**	**.10** ^**^	0.94	3.94 (1, 55)	.052	.07**	**0.94**	**4.03 (1, 60)**	**.049**	**.06** ^**^	**0.92**	**5.36 (1, 59)**	**.024**	**.08** ^**^

Notes

* = Small effect

** = Medium effect

*** = Large effect per Cohen (1998) guidelines. **Bold font** denotes **significant moderation effect**, significant main effects of time on each outcome are not bolded. CSE = Coping self-efficacy; Time = Change in pre-to-post program scores;; η_p_^2^ = Partial eta-squared; A total of our separate models were run for each CSE subscale, each having a time main effect and one interaction term.

**Fig 2:**
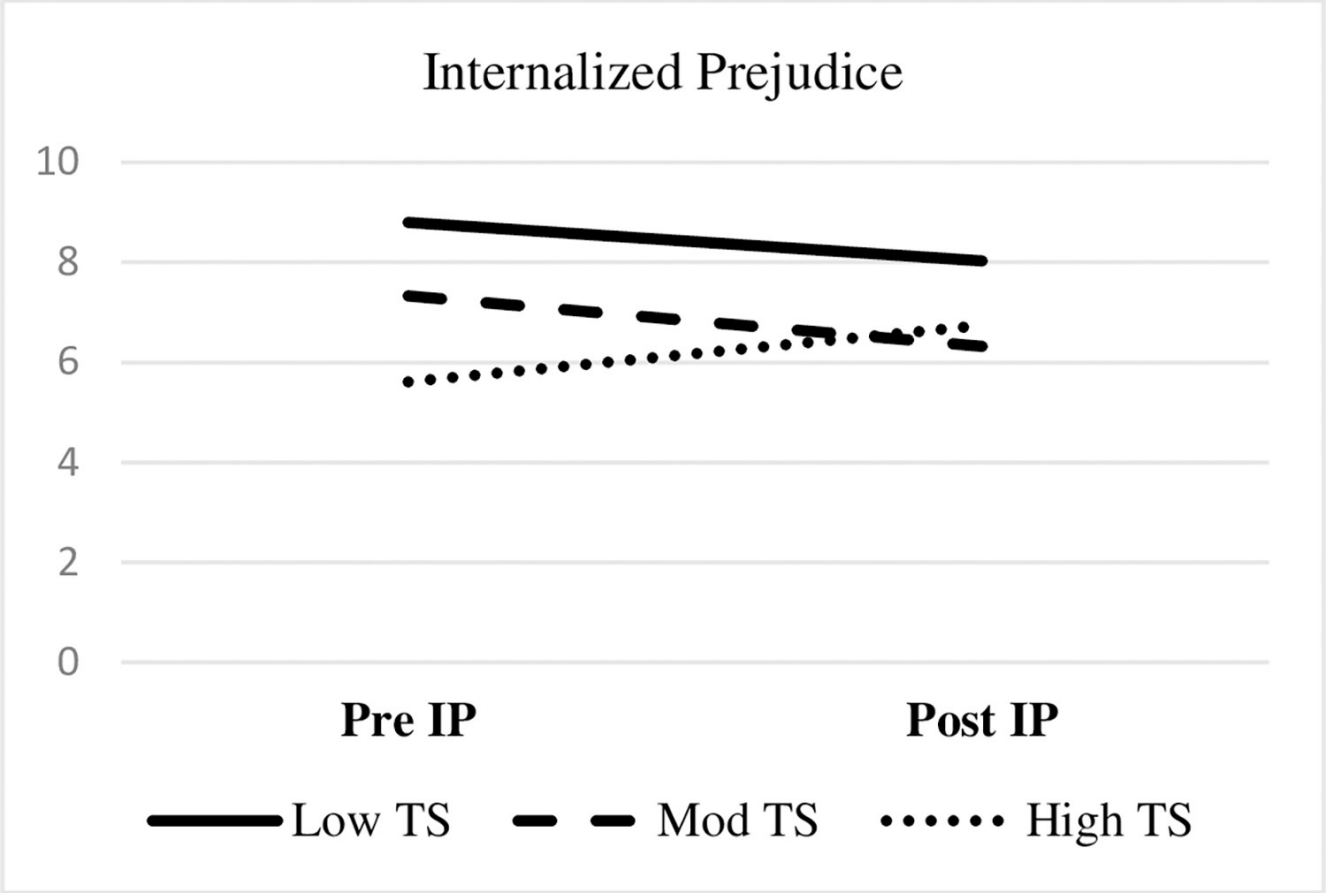
Thought stopping coping-self efficacy as a significant moderator of internalized prejudice. Notes. TS = Thought stopping subscale of the Coping Self-Efficacy scale; IP = internalized prejudice subscale of the Lesbian, Gay, and Bisexual Identity Scale (LGBIS). Test statistics presented in Table 3.

Referencing Table 3, thought stopping CSE moderated pre-post reductions in internalized prejudice (medium effect). Fig 2 depicts the thought stopping CSE by internalized prejudice interaction. LGBTQ+ veterans with moderate thought stopping CSE reported reductions in internalized prejudice. An unanticipated pattern emerged in that LGBTQ+ veterans with high thought stopping CSE reported increased internalized prejudice.

**Fig 3:**
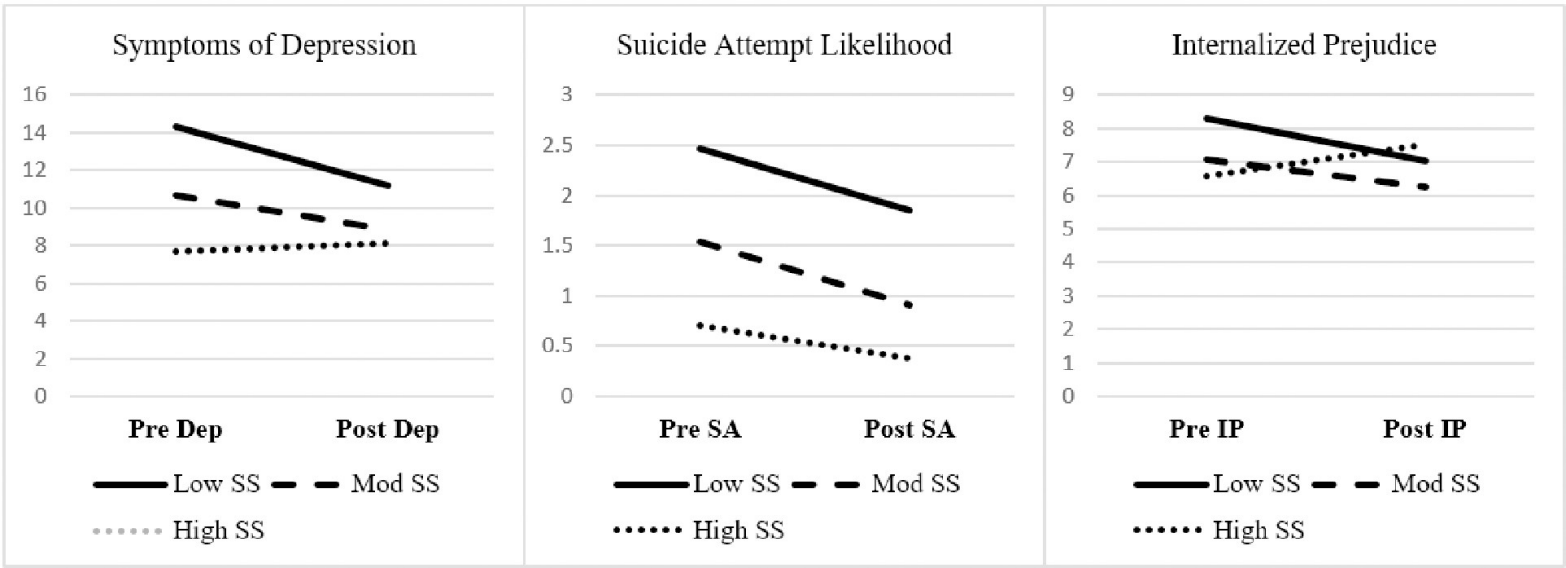
Social support coping-self efficacy as a significant moderator of symptoms of depression, suicidality, and internalized prejudice. Notes. Dep = Symptoms of depression; SA = suicidality; IP = internalized prejudice subscale of the Lesbian, Gay, and Bisexual Identity Scale (LGBIS); SS = Social support subscale of the Coping Self-Efficacy scale. Test statistics in Table 3.

Also in Table 3, social support CSE moderated pre-post reductions in depressive symptoms, suicidality, and internalized prejudice (all medium effects). Fig 3 depicts the interactions. In line with expectations, LGBTQ+ veterans low in social support CSE reported greater reductions in depressive symptoms, suicidal attempt likelihood, and internalized prejudice after attending the group. Also, LGBTQ+ veterans moderate in social support CSE reported reductions in depressive symptoms, suicidal attempt likelihood, and internalized prejudice. An unanticipated pattern emerged in that LGBTQ+ veterans high in social support CSE reported increased internalized prejudice.

### Demographics as moderators of PRIDE in All Who Served program outcomes

**Fig 4:**
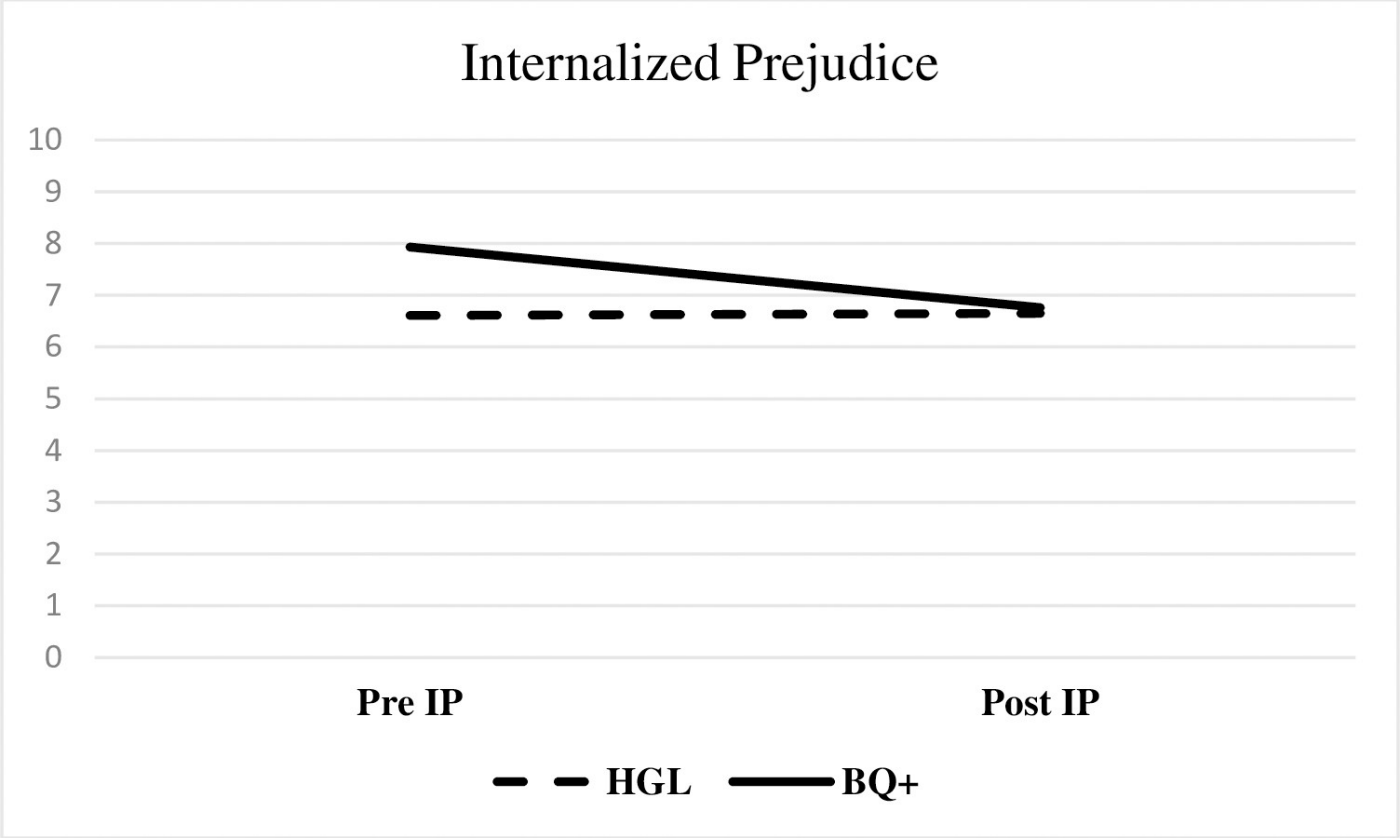
Sexual orientation as a significant moderator of internalized prejudice. Notes. IP = internalized prejudice subscale of the Lesbian, Gay, and Bisexual Identity Scale (LGBIS); HGL = Heterosexual/gay/lesbian; BQ+ = Bisexual/queer/questioning/something else. Test statistics in Table 4.

Table 4 contains demographic moderation analysis test statistics. The only observed significant moderation effect was sexual orientation on pre-post reductions in internalized prejudice (medium effect). Fig 4 depicts this interaction. Heterosexual, gay, and lesbian veterans reported no change in internalized prejudice, whereas veterans who identified as bisexual or a related identity reported reductions in internalized prejudice.

**Table 4:** Moderation analyses using repeated measures general linear modeling for demographic characteristics.

	Depressive Symptoms				Anxiety Symptoms				Suicidality				Internalized Prejudice			
Predictor	Wilks’ λ	F (df)	*p*-value	η_p_^2^	Wilks’ λ	F (df)	*p*-value	η_p_^2^	Wilks’ λ	F (df)	*p*-value	η_p_^2^	Wilks’ λ	F (df)	*p*-value	η_p_^2^
Time	0.94	3.32 (1, 54)	.074	.06**	0.97	1.88 (1, 54)	.176	.03*	0.93	4.46 (1, 59)	.039	.07**	0.98	1.25 (1, 58)	.268	.02*
Age by time	0.98	1.29 (1, 54)	.260	.02*	0.99	0.46 (1, 54)	.500	.01*	0.98	1.29 (1, 59)	.261	.02*	0.99	0.31 (1, 58)	.579	.005*
Time	0.88	7.52 (1, 55)	.008	.12**	0.88	7.75 (1, 55)	.007	.12**	0.82	13.14 (1, 60)	.001	.18***	0.93	4.38 (1, 59)	.041	.07**
Race by time	0.94	3.34 (1, 55)	.073	.06**	0.94	3.35 (1, 55)	.073	.06**	0.99	0.36 (1, 60)	.553	.01*	1.00	0.08 (1, 59)	.778	.001*
Time	0.87	8.05 (1, 54)	.006	.13**	0.88	7.07 (1, 54)	.010	.12**	0.82	12.57 (1, 59)	.001	.18***	0.91	5.78 (1, 58)	.019	.09**
Sexual Orientation by time	0.96	1.95 (1, 54)	.169	.03*	1.00	0.01 (1, 54)	.936	.00*	0.99	0.43 (1, 59)	.513	.01*	**0.91**	**5.36 (1, 58)**	**.024**	**.08** **
Time	0.89	6.08 (1, 52)	.017	.10**	0.88	6.93 (1, 52)	.011	.12**	0.83	11.93 (1, 57)	.001	.17***	0.95	3.10 (1, 56)	.084	.05*
Gender Identity by time	0.97	0.68 (1, 52)	.511	.03*	1.00	0.06 (1, 52)	.940	.00*	0.98	0.53 (1, 57)	.593	.02*	0.98	0.57 (1, 56)	.567	.02*

Notes

* = Small effect

** = Medium effect

*** = Large effect per Cohen (1998) guidelines. **Bold font** denotes **significant moderation effect**. Race = White or racial minority; Sexual orientation = Heterosexual/gay/lesbian or bisexual/queer/questioning/other; Gender = Cisgender male, cisgender female, or transgender/gender diverse; Time = Change in pre-to-post program scores at 10 weeks; η_p_^2^ = Partial eta-squared. A separate model was run for each CSE subscale, such that each has a main effect of time (pre-post group change) and one interaction term.

## Discussion

The Pride in All Who Served intervention is more impactful for veterans with low coping self-efficacy than those with high coping self-efficacy. Specifically, LGBTQ+ veterans possessing low, and at times moderate, levels of CSE experienced the largest improvements in mental health and internalized stigma after the 10-week intervention. LGBTQ+ veterans with low or moderate problem-solving CSE reported reductions in symptoms of depression, anxiety, and internalized prejudice. Moreover, LGBTQ+ veterans with low or moderate degrees of social support CSE saw reductions in symptoms of depression, internalized prejudice, and suicidality. The moderating role of coping-related beliefs is consistent with the Minority Stress Model [10,11] premise that coping and connectedness can buffer identity- and discrimination-related impacts on mental health and well-being. Our findings are also consistent with cross-sectional research showing (a) stress-buffering effects of coping (e.g., using social support) among LGBTQ+ people [25,26] and (b) a pattern of negative associations between CSE beliefs and severity of symptoms of mental illness among LGBTQ+ people [40,41].

From a CSE perspective, our findings are not consistent with preliminary tests of CSE and suicidality among active-duty military personnel. In particular, Cunningham and colleagues [42] identified CSE thought stopping beliefs as a primary factor implicated in suicide risk. Bowling and colleagues [64] observed that (a) transgender and non-binary adults possessed lower CSE thought stopping compared to other genders and (b) all sexual minority adults in their sample demonstrated lower CSE thought stopping compared to heterosexual counterparts. Prior research therefore illustrates the potential primacy of CSE thought stopping in understanding suicide among military personnel, and between-groups variation by LGBTQ+ identity. On the contrary, we observed primacy of CSE problem-solving and social support for LGBTQ+ veterans, suggesting that varying aspects of CSE may be more beneficial or relevant to cisgender/heterosexual versus LGBTQ+ military-affiliated personnel.

CSE problem-solving and social support belief moderation patterns raise the question of possible mechanisms by which these findings can be contextualized. The PRIDE in All Who Served program [2] is one of a broader set of affirmative group-based interventions for LGBTQ+ persons emerging in the literature [65,66]. Recent qualitative inquiries into the benefits of affirmative cognitive behavior therapy (CBT)-based group interventions in particular [67,68] may shed light on why low-to-moderate CSE beliefs are associated with PRIDE program gains. Sexual minority persons participating in affirmative CBT group interventions have suggested that engagement with CBT tools and concepts [68], as well as the decreased sense of loneliness from interaction with other sexual minority men of color [67], may explain improved mental health. Applied to our findings, LGBTQ+ veterans with lower levels of CSE social support and problem-solving beliefs (a CBT coping skill) may have experienced gains in these areas, thereby affecting positive well-being outcomes. Future research could be conducted to engage group participants in mixed-method inquiry around plausible mechanisms of action to refine our understanding for future PRIDE implementation and other health promotion interventions.

Our CSE moderation analyses showed some indication that those high in CSE thought stopping and social support beliefs may experience slight increases in internalized prejudice. This pattern is contrary to what we expected. While this pattern should be probed for further understanding, we also avoid over emphasis of the trend, particularly given the low levels of internalized prejudice reported at both pre- and post-group timepoints. At least two plausible explanations exist. First, to the extent those high in CSE beliefs at intervention baseline are also quite low in internalized prejudice, the pattern over the course of the PRIDE program may reflect some regression to the mean. Alternatively, characteristics of the group may have affected levels of internalized prejudice. Lloyd and colleagues [68] observed that some sexual minority adults taking part in an adapted CBT group therapy program found generational divides between participant groups to harm group cohesion.

Overall, demographics did not moderate program outcomes, suggesting that an inclusive health promotion group–as originally developed–is likely a good match for the heterogenous individuals typically enrolled. One notable exception was observed for veterans who reported their sexual orientations as bisexual, queer, questioning or something else (e.g., pansexual, see Table 4). Veterans with bisexual and related identities in this sample reported greater reduction in internalized prejudice following the group than their single-gender attracted peers (i.e., heterosexual, gay, or lesbian veterans). Though based on a relatively small sample and conducted within a clinical setting rather than a rigorous clinical trial, the data presented here offer preliminary evidence of an intervention with a promising impact for individuals with bisexual, queer, and related identities consistent with recent calls to action for the field [69]. The unique experiences of discrimination, dismissal, and invisibility of bisexual people including lack of in-group community support has been reported in both qualitative and larger quantitative/epidemiological studies [70,71]. For example, individuals who are attracted to more than one gender may experience discrimination from both heterosexual individuals as well as from within the LGBTQ+ community.

### Limitations & opportunities for future work

Our findings are of course contextualized by several limitations. First, the limited number of individuals in the current sample requires that future work replicate these preliminary findings. We elected to disaggregate LGBTQ+ individuals in our moderation analyses into categorical groups that would not be combined had the sample sizes of subgroups been adequate for comparisons. For example, combining individuals who identified as heterosexual and lesbian/gay in order to examine impacts for individuals that identify as bisexual or a related identity (e.g., pansexual, queer) is not ideal. Oversampling of veterans who identity as bisexual and related identities will be required to replicate the preliminary findings we observed. Importantly, all of the participants in this sample did identify as LGBTQ+; thus the heterosexual-identified participants included likely experience minority stress comparable to rest of the sample (e.g., a transwoman that is attracted to men and identifies as heterosexual). Similarly, combining transgender and gender diverse individuals into a single group to enable gender identity comparisons and collapsing across racial categories would not be required in larger samples. Another limitation is the attrition from pre- to post-assessment and the limited time frame for follow-up (e.g., outcomes at 10-weeks). Expansion to include more individuals who identity as racial and ethnic minorities, a larger sample that would permit more in-depth analyses of gender [57] and other demographics, and exploration of the longevity of these findings (e.g., after 3-months or 12-months) is necessary in future work. More rigorous designs that include comparison conditions would also increase confidence in outcomes for the Pride in All Who Serve program and related services.

## Conclusions

As more healthcare services are developed to address health inequities for historically marginalized people, papers like this one may advance understanding of individual characteristics (e.g., coping self-efficacy, bisexual and related identities) not widely considered in clinical care. This paper provides an initial examination of potential moderators of treatment outcomes for LGBTQ+ veterans attending a health promotion group at VA Medical Centers in the US that may inform future tailoring of programs that build resilience, nurture a sense of community, and ultimately provide tangible, upstream suicide prevention strategies. Although the absence of moderation in a small sample is not conclusive, it provides preliminary program evaluation data to clinicians and policy makers implementing programs for LGBTQ+ veterans. For example, clinical settings may be tempted to separate LGBQ and transgender/non-binary individuals into separate groups. In our evaluation of this health promotion group, this type of pre-determined segregation may come with risks. For example, veterans who have not historically identified as gender diverse who are referred to a group that omits topics related to gender identity could lose the opportunity to reflect on gender outside of a gender binary. Anecdotally, clinicians delivering the group also report rich discussions between younger and older veterans, those with different racial identities (e.g., Black and White individuals) and along other intersections of identity (e.g., religious affiliation, geographic rurality) in the same group. Overall, this study provides an understanding of how LGBTQ+ Veterans with fewer coping resources can especially benefit from the PRIDE in All Who Served program.
